# The association between malaria and malnutrition among under-five children in Shashogo District, Southern Ethiopia: a case-control study

**DOI:** 10.1186/s40249-016-0221-y

**Published:** 2017-01-13

**Authors:** Terefe Gone, Fiseha Lemango, Endale Eliso, Samuel Yohannes, Tadele Yohannes

**Affiliations:** 1 Department of Medical Laboratory Sciences, Hosanna College of Health Sciences, P.O. BOX 159, Hosanna, Ethiopia; 2 Department of Public Health, Hosanna College of Health Sciences, Hosanna, Ethiopia; 3 Shashogo District Health Office, Shashogo, Ethiopia

**Keywords:** Malaria, Malnutrition, Under-five children, Ethiopia

## Abstract

**Background:**

Recent studies have presented conflicting findings about whether malaria is associated with an increased or decreased risk of malnutrition. Therefore, assessing the relationship between these two disastrous diseases in the most vulnerable groups, such as in children aged below 5 years (under-five children), may lead to the discovery of new low-cost and effective aides to current methods of malnutrition prevention in malaria-endemic areas. Therefore, this study was conducted to assess the relationship between malaria and malnutrition among under five children in an area with a high degree of malaria transmission.

**Methods:**

The study involved comparing malnourished children aged 6–59 months and nourished children of the same age for their past exposure to malaria, in Shashogo District, Southern Ethiopia. A validated structured questionnaire was used to collect home to home socioeconomic data and anthropometric instruments for clinical data. The collected data were analysed using descriptive and inferential statistics by means of EpiData entry software and STATA data analysis software.

**Results:**

A total of 356 (89 malnourished and 267 nourished) under-five children participated in the study. Previous exposure to *Plasmodium* infection was found to be a predictor for the manifestation of malnutrition in under-five children (*P* = 0.02 [*OR* = 1.87, *CI* = 1.115–3.138]). Children from a household with a monthly income of less than USD 15 were 4.5 more likely to be malnourished as compared to the other children (*P* = 0.001 [*OR* = 0.422, *CI* = 0.181–0.978]).

**Conclusion:**

This study found that exposure to *Plasmodium* has a significant impact on the nutritional status of children. In addition, socio-demographic factors, such as family income, may play a role in determining whether children are malnourished or not and may lead to increased morbidity due to malnourishment in children living in malaria-endemic areas. Therefore, malnutrition control interventions should be consolidated with malaria prevention strategies particularly in high malaria transmission areas.

**Electronic supplementary material:**

The online version of this article (doi:10.1186/s40249-016-0221-y) contains supplementary material, which is available to authorized users.

## Multilingual abstracts

Please see Additional file 1 for translations of the abstract into the five official working languages of the United Nations.

## Background

Malaria and under nutrition are the two major causes of childhood mortality in Sub-Saharan Africa (SSA). Each year, malaria kills more than 800 000 people, of which 91% reside in Africa and 85% are children aged below 5 years (under-five children) [1, 2]. Meanwhile, under nutrition is considered to be the underlying cause for more than 50% of deaths of under-five children in Sub-Saharan Africa. In Africa, malnutrition is highly prevalent: 39, 8 and 28% of under-five children are stunted, wasted or underweight, respectively [3, 4].

In Ethiopia, malnutrition and malaria are the top causes of morbidity and mortality in under-five children [2, 5]. The country has the second highest rate of malnutrition in SSA [6]. According to Ethiopia’s 2011 demographic and health survey, the prevalence of underweight, stunting and wasting was very high: 29, 44 and 10%, respectively, for the nation as a whole, and 28.3, 44.1 and 7.6% for the Southern Nations, Nationalities and Peoples’ Region (SNNPR) [5]. Out of all the febrile diseases that under-five children had nationally in 2011, 19.7% were infected with malaria [7].

Although malaria and malnutrition frequently coexist [8], limited studies have been done evaluating the effect of malaria on malnutrition, and when such studies have been carried out, the results have been contradictory. Some studies have reported that children with previous exposure to malaria have a higher risk of becoming malnourished, as characterised by either stunting, underweight or wasting. Other studies have reported a lower risk, and others have reported no association between malaria and malnourishment at all [9, 10].

Infection with *Plasmodium falciparum* or *P.vivax*, the two predominant *Plasmodium* species in Ethiopia, has been associated with impaired physical growth in children [9–11]. Catch-up growth has been observed following interventions focusing on disease prevention, which suggests that malaria plays a role in the aetiology of malnutrition and contributes to the downward cycle of impaired development of mental functions [12–14].

Given that the relationship between malaria and malnutrition is complex, the individual impacts of the diseases, as well as their combined impact, on under-five children is enormous in SSA. Hence, understanding the relationship between these two diseases is of great public health importance. In addition, relatively few studies have examined the association between malaria with malnutrition in highly endemic malaria areas in SSA, particularly in Ethiopia [15].

The World Health Organization (WHO) Integrated Management of Childhood Illness initiative is based on the premise that combining efforts to promote the appropriate case management of serious infectious diseases such as malaria with nutritional interventions, immunisation programmes, and other disease prevention and health promotion activities will be more effective in decreasing child mortality than implementing any one of the components separately [16, 17]. In other words, if malaria increases the risk of malnutrition, intervention programmes that succeed in preventing and controlling malaria may have potential for enhancing the survival of children in regions with malaria endemicity. Therefore, this study was conducted to assess the relationship between malaria and malnutrition among under five children in an area with a high degree of malaria transmission.

## Methods

### Study area

**Fig. 1:**
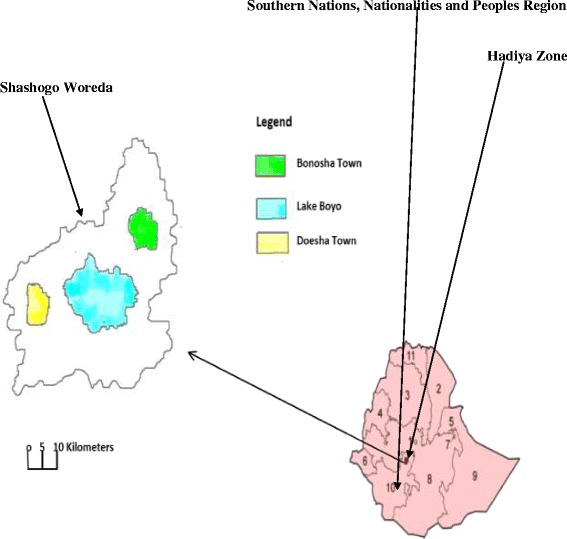
Map of the study area

The study was conducted from May to June 2015 in Hadiya Zone, Shashogo district, which is situated 224 km from the capital Addis Ababa, 117 km from Hawassa, the capital of SNNPR, and 52 km from the zonal capital Hosanna. It is positioned at an elevation ranging from 1800 to 2 000 m above sea level, and lies between 81^0^97′50″– 82^0^5′60″ N latitude and 39^0^80′10″– 40^0^28′00″ E longitude (see Fig. 1). In Shashogo, there are 36 *kebeles* (34 rural and two urban) within an area of 32 310 km^2^. The district has a total population of 127 281, of which 20 460 are under-five children [18]. The area has predominantly dry *kola* (hot low land) agro ecology. The rainfall pattern is bimodal: the months from May to September are marked by a relatively higher rainfall, while the months from November to February are dry. The long rainy season is between June and September, during which crop cultivation takes place. The total annual rainfall reaches 1 005.1 mm. The mean maximum daily temperature is 21.6 °C (February), while the mean minimum daily temperature is 18.5 °C (July) [National Meteorological Agency, Hawassa Directorate]. Water bodies such as streams and rivers are common in the area. There is also a lake, which surrounds two of the *kebeles* and acts as a potential mosquito-breeding site, particularly during the dry season.

The district has five health centres and 36 health posts each with two health extension workers. The district’s health service coverage reached 98% in 2013. The main malaria prevention and control strategies include insecticide-treated bed nets (ITNs), indoor residual spraying, use of a larvicidal chemical (Abate®), environmental modification and case management through early detection and treatment. Malnutrition control strategies include community-based nutrition, integrated community case management, integrated management of illness in newborns and children, and essential nutritional actions [Unpublished report from the District Health Office].

### Study design

A community-based case-control study was carried out.

The inclusion criteria were: being between the ages of six and 59 months and being a resident of the study area for more than 6 months.

The exclusion criteria were: presence of a detectable handicap that might alter linear growth. If parents of selected children withdrew informed consent or were absent during the household visit, those children were replaced. For controls, children fulfilling the inclusion criteria from the next household took their place, and for cases, children from the next house number on the malnutrition registration book in the respective health posts took their place.

Cases were defined as those under-five children who were stunted or wasted, i.e. having a height-for-age Z-score (HAZ) or a weight-for-age Z-score (WHZ) < -2, respectively (as calculated by subtracting mean value of height or weight of the specific population from the individual value divided by the standard deviation of the population). Similarly, controls were defined as well-nourished under-five children, i.e. having a HAZ or WHZ > -2.

### Sample size estimation and sampling technique

The formula for the estimation of a sample size for an independent case-control study [19] was used taking 48% exposure to malaria in controls from a previous study conducted in Kenya [20], and taking into account a 95% confidence interval (*CI*) (1.96), 5% level of significance (α), power of 80% (0.84) and 1:3 controls to cases ratio (*r* = 3) to detect an odds ratio of 2.0 or greater, it was determined that a minimum of 89 study subjects were required for cases and 267 were required for controls. Thus, 89 children were randomly selected by a lottery method from a list of 186 registered malnourished children who were being followed up by health extension workers in each *kebele* during the study period. Only one child, either nourished or malnourished, was selected from a single household. Controls were also selected by a lottery method, if there was more than one under-five child in a household. Therefore, 89 malnourished children and 267 well-nourished children aged 6–59 months were recruited.

### Data collection methods

A structured questionnaire was used to extract information on the socio-economic status and educational status of the caregiver. The ages of the children were taken from their vaccination cards (if available) or from the verbal report given by the caregiver. The year and month of birth were determined for all children. For those children with unknown days of birth, the 15^th^ of the month was recorded.

In accordance with internationally accepted practices [21], anthropometric parameters such as weight and height were recorded for both cases and controls to confirm the health posts’ records for the cases and to determine whether the controls were in fact nourished.

Weight was measured using a spring scale for younger children (up to approximately 2 years of age) or with a standing scale for older children (Seca, Hamburg, Germany); both precise to the nearest 10 g. Recumbent length measurements were taken using a stadiometer for children up to approximately 2 years of age. For children older than two, standing height measurements were taken. All length and height measurements were precise to the nearest 1 cm. The 2006 WHO growth reference standards were used to calculate HAZ, WHZ and weight for-age Z-score (WAZ) [22]. Children were classified as stunted or wasted if the HAZ or WHZ was < -2, respectively.

Data collectors, assistants and supervisors were trained on basic data collection and supervisory techniques, and hence supervisors regularly checked the performance of the data collectors in terms of accuracy, completeness and appropriateness. Principal investigators gave feedback on the activities of the previous day to both data collectors and supervisors.

Parents or caregivers were asked about their child’s malaria history. Both nourished and malnourished children with a history of malaria were checked for laboratory confirmation of malaria in the health institutions where they had been microscopically diagnosed within up to a year prior to the study commencing. Children whose malaria diagnosis was not confirmed by laboratory confirmation were replaced by children from neighbouring households who fulfilled the inclusion criteria.

### Data analysis

The data obtained from the study were computerised using EpiData version3.1 software and exported to statistical STATA version 11 software for analysis. Means and standard deviations were calculated for continuous variables. Odds ratios (*OR*s with 95%*CI*s) were used to measure the strength of the statistical associations between the outcomes and exposures sing the binary logistic regression model and multivariable logistic regression analysis. All variables were initially tested for associations between malnutrition and malaria using the binary logistic regression model. Variables that showed a statistical association were then analysed using multivariate logistic regression to check if the association still existed after controlling for possible confounders. All statistical tests and generalisations were performed assuming a 95%*CI* and 5% level of significance.

### Ethical considerations

The study obtained ethical clearance from the Ethical Research Committee of the Hosanna College of Health Sciences. Written consent was also secured from the Shashogo administration and written informed assent was obtained from the heads of households or the child’s caregiver. Children whose anthropometric indicators showed that they were malnourished were reported to the nearest heath facility for further diagnosis and treatment (that is, if they were not already recorded in the health post as being malnourished).

## Results

### Socio-demographic characteristics

A total of 356 (89 malnourished and 267 nourished) under-five children participated in the study. Of these, 196 (55.1%) were females and the remaining 160 (44.9%) were males. One hundred and 26 (35.4%) participants were in the age range of 26–38 months and 107 (30.1%) were in the age range of 13–25 months. Almost half (48.0%) of the children were from households with more than six family members. 300 and 33 (93.5%) parents (caregivers) were married, and 69.9% had never obtained formal education and hence could not read or write. Almost three-quarters (73.6%) of the caregivers were housewives, followed by farmers (23.0%). The mean monthly income of the families was 377.6 Ethiopian Birr (USD 17.3) (see Table 1).

**Table 1:** Socio-demographic characteristics and history of malaria in malnourished and nourished under-five children in Shashogo, Southern Ethiopia, 2015

Variables		Frequency (%)	Malnourished *n* (%)	Nourished *n* (%)	Crude OR (95%CI)	*P*-value
Age, in months	6–12^a^	74 (20.8)	21 (28.4)	53 (71.6)	1 (0.603–1.654)	0.06
	13–25	107 (30.1)	37 (34.6)	70 (65.4)	1.39 (0.936–2.07)	
	26–38	126 (35.4)	20 (15.9)	106 (84.1)	0.505 (0.316–0.806)	
	39–59	49 (13.7)	11 (22.5)	38 (77.5)	0.819 (0.427–1.57)	
Sex	Male^a^	160 (44.9)	45 (28.1)	115 (71.9)	1 (0.712–1.400)	0.17
	Female	196 (55.1)	44 (22.4)	152 (77.6)	0.716 (0.514–1.00)	
Family size	<4^a^	49 (13.8)	12 (24.5)	37 (75.5)	1 (0.532–1.89)	0.35
	4–6	136 (38.2)	28 (20.6)	108 (79.4)	0.751 (0.499–1.330)	
	>6	171 (48.0)	49 (28.7)	122 (71.3)	1.144 (0.823–1.593)	
Family monthly income	<USD 15^a^	188 (52.8)	61 (32.4)	127 (67.6)	1 (0.744–1.364)	0.0001*
	USD 15–25	98 (27.5)	21 (21.4)	77 (78.6)	0.578 (0.360–0.93)	
	≥USD 25	70 (19.7)	7 (10.0)	63 (90.0)	0.222 (0.100–0.484)	
Marital status of the caregiver	Married^a^	333 (93.5)	78 (23.4)	255 (76.6)	1 (0.762–1.317)	0.763
	Single	3 (0.8)	1 (33.3)	2 (66.7)	0.730 (0.421–1.265)	
	Divorced	6 (1.7)	2 (33.3)	4 (66.7)	5.464 (1.000–29.833)	
	Widowed	10 (2.8)	3 (30.0)	7 (70.0)	-------	
	Separated	4 (1.2)	2 (50.0)	2 (50.0)	2.732 (0.385–19.396)	
Literacy/ educational status of the caregiver	Cannot read or write	249 (69.9)	70 (28.1)	179 (71.9)	1 (0.756–1.31)	0.066
	Can read or	16 (4.5)	2 (33.3)	14 (66.7)	0.349 (0.078–1.534)	
	Primary (1–8)	84 (23.6)	16 (19.0)	68 (81.0)	0.620 (0.363–1.054)	
	Secondary and above	7 (2.0)	1 (14.3)	6 (85.7)	0.407 (0.049-3.376)	
Occupation of the caregiver	Housewife^a^	262 (73.6)	73 (27.7)	189 (72.1)	1 (0.756–0.293)	0.085
	Farmer	82 (23.0)	16 (19.5)	66 (80.5)	0.659 (0.381–1.11)	
	Student	9 (2.5)	0 (0)	9 (100.0)	-----	
	Pprivate worker	3 (0.9)	0 (0)	3 (100.0)	------	
Religion of the caregiver	Christian^a^	220 (61.8)	49 (22.3)	171 (77.7)	1 (0.732–1.368)	0.107
	Muslim	132 (37.1)	38 (28.8)	94 (71.2)	1.391 (0.957–2.02)	
	Other	4 (1.1)	2 (50.0)	2 (50.0)	3.311 (0.467–23.507)	
Previous malaria status	Negative^a^	254 (71.4)	52 (20.5)	202 (79.5)	1.00 (0.742–1.346)	0.016*
	Positive	102 (28.6)	37 (36.3)	65 (63.7)	1.96 (1.304–2.940)	
Type of *Plasmodium* species	*P. falciparum* ^*a*^	36 (35.3)	10 (27.8)	26 (72.2)	1.00 (0.481–2.073)	0.22
	*P. vivax*	66 (64.7)	27 (40.9)	39 (59.1)	1.73 (1.055–2.844)	
Elapsed time since last malaria onset	2–5 months^a^	63 (61.8)	22 (34.9)	41 (65.1)	1.00 (0.596–1.678)	0.67
	6–12 months	39 (38.2)	14 (35.9)	25 (64.1)	0.97 (0.484–1.953)	

*Statistically significant when *P* < 0.05

^a^reference category

### Analysis of the malnourished cases

Of the 89 malnourished children, 81 (91.0%) were wasted and the rest (9.0%) were stunted. Of these, 11.3% had severe malnutrition (HAZ or WAZ < -3). A significant proportion (41.6%) of malnourished children were aged between 13 and 25 months and about a quarter were aged between 26 and 38 months (22.5%). However, the association between age and malnutrition was not statistically significant. There were slightly more malnourished male children than female malnourished children participating in the study, i.e. 51 and 49%, respectively.

Remarkably, more than half (55.1%) of the malnourished children were from a family with more than six members. Similarly, 72 (80.9%) cases had caregivers who did not have any formal education. However, neither family size (*P* = 0.35) nor the caregiver’s educational status (*P* = 0.06) was significantly associated with malnutrition.

In terms of family income, 61 (68.5%) children were from a house hold that had a monthly income of less than 300 Ethiopian Birr (USD 13.8). The association between a family’s monthly income and malnutrition was found to be statistically significant by both binary and multivariate logistic regression analyses. In other words, children from a household with a monthly income of less than USD 15 were 4.5 times more likely to be affected by malnutrition as compared to the other children (*P* = 0.001 [*OR* = 0.422, *CI* = 0.181–0.978]) (see Table 2).

**Table 2:** Multivariate analysis showing statistically significant variables between malaria and malnutrition among under-five children in Shashogo, Southern Ethiopia, 2015

Variables		Frequency (%)	Malnourished *n* (%)	Nourished *n* (%)	Adjusted OR (95%CI)	*P*-value
Previous malaria status	Negative^a^	254 (71.4)	56 (22.0)	198 (78.0)	1.87 (1.12–3.14)	0.02*
	Positive	102 (28.6)	36 (35.3)	66 (64.7)		
Family monthly income	<USD 15^a^	188 (52.8)	61 (32.4)	127 (67.6)	0.422 (0.181–0.978)	0.0001*
	USD 15–25	98 (27.5)	21 (21.4)	77 (78.6)		
	≥USD 25	70 (19.7)	7 (10.0)	63 (90.0)		

*Statistically significant when *P* < 0.05

^a^reference category

### Association between malaria and malnutrition

Previous exposure to *Plasmodium* infection was found to be a predictor for the manifestation of malnutrition in under-five children (*P* = 0.02 [*OR* = 1.87, *CI* = 1.115–3.138]), i.e. children previously exposed to malaria were 1.87 times more likely to be malnourished than children unexposed to malaria (see Table 2). Malnutrition was higher in children with a history of *P. Vivax* infection (40.9%) than in those with a history of *P. falciparum* (27.8%) infection, but the difference was not statistically significant (*P* = 0.22).

## Discussion

Whether there is an association between malaria and malnutrition is a controversial issue, in that some studies report that children exposed to malaria have a higher risk of becoming malnourished [12–14], while others have found that no association exists [15]. This study did find a statistically significant association between malaria and malnutrition (*P* < 0.05). Under-five children previously exposed to *Plasmodium* infection were found to be 1.87 times more likely to develop malnutrition than non-exposed children. These findings are similar to those of many other studies, which have reported that malaria can affect the nutritional status of children. A study conducted in the rural community of Amazonian region indicated that children who suffered malaria episodes presented worse anthropometric parameters [23]. Likewise, in a study carried out in a holoendemic malaria area of Tanzania, catch-up growth was seen in those children who used ITNs [12], which suggests that *Plasmodium* infection plays a role in the aetiology of malnutrition. However, the findings of the current study are contrary to a community-based study conducted in Southwest Ethiopia, which reported that there is no association between malaria and under nutrition [15].

This study did not find a significant association between malnutrition and type of malaria species, i.e. *P. vivax* and *P. falciparum*, the two dominant *Plasmodium* species in the study area and the country in general [9]. However, there were a significantly larger proportion of malnourished children who were infected with *P. vivax* than with *P. falciparum*. Similarly, different studies showed that infection with *P. vivax* was associated with a greater risk of malnutrition compared with a *P. falciparum* infection [24].

Even though there was no statistically significant association observed between age and malnutrition, there were a higher proportion of malnourished children in the age group of 13–25 months. This is in accordance with studies conducted in Ethiopia and elsewhere in Africa [25–28], which have reported that children in the older age group (26+ months) are at a significantly lower risk of being malnourished compared to the younger age groups. The younger age groups, particularly the age range of 13–25 months, might be at a greater risk of being malnourished due to the increased nutritional needs for growth and development that this age group requires, or could be due to lack of a balanced diet, and meal frequency. Furthermore, lack of a diet that a child prefers might increase the risk of being malnourished since they may not comfortably take the other food stuffs [29].

This study also found that the risk of malnutrition significantly increases as the family income decreases, particularly when it reaches less than USD 15. Children from a family with monthly income of less than USD 15were found to be 4.5 times more prone to malnutrition than those from a family with a higher monthly income. In agreement with this, a facility-based study conducted in Gondar, Northern Ethiopia [30] showed that the risk of severe acute malnutrition increased when the monthly income was lower than USD 50. This finding is also supported by studies done in Western Ethiopia [31], and other African countries such as Nigeria, Sudan and Zimbabwe [32–34].

Parental illiteracy and large family size are the two most reported socio-demographic characteristics, which, in one way or another, may significantly affect a child’s nutritional status. In a case-control study conducted in Bangladesh, maternal illiteracy was associated with a fourfold increase in the risk of children acquiring severe acute malnutrition [35]. However, in the present study these two characteristics were not found to be significantly associated with malnutrition, even though more than three-quarters of the malnourished cases in this study were cared for by individuals without any formal education and more than half of the cases were from a household with more than six family members. The absence of a statistical association between parental illiteracy and larger family size with malnutrition in this study might possibly be due to the fact that most of the study participants had similar family sizes and caregivers with similar educational statuses, which might have hindered detecting whether actual differences in nutritional statuses existed.

This study had some limitations. A major one was that some potential confounders such as diarrhoeal diseases, parasitic diseases and other agents were excluded from this study. The other limitation is that a record review was used to identify previous malaria exposure which might lack concreteness. In addition, the study merely analysed the relationship between malaria and malnutrition not the mechanism through which plasmodium depletes nutrients.

## Conclusions

The present study revealed that exposure to *Plasmodium* infection has a significant impact on the nutritional status of under-five children, particularly in malaria-endemic areas. The study also shown that socio-demographic factors such as family income can also play a role in worsening morbidity due to malnutrition. Therefore, malnutrition control interventions should be consolidated with malaria prevention strategies in malaria-endemic areas. In these areas, increasing access to education, providing more job opportunities and making people more aware about family planning methods so that they can access a balanced diet and be aware of how to feed their children.

## Additional file

Additional file 1: Multilingual abstracts in the five official working languages of the United Nations. (PDF 849 kb)

## Abbreviations

CI: Confidence interval
HAZ: Height-for-age Z-score
ITN: Insecticide-treated bed net
OR: Odds ratio
SNNPR: Southern Nations, Nationalities and Peoples’ Region
SSA: Sub-Saharan Africa
USD: United States dollar
WAZ: Weight-for-age Z-score
WHO: World Health Organization
WHZ: Weight-for-height Z-score
