# Supplementary material for: The association between malaria and malnutrition among under-five children in Shashogo District, Southern Ethiopia: a case-control study
Source: Infect Dis Poverty. 2017 Jan 13;6:9. doi: 10.1186/s40249-016-0221-y (PMC5234126; doi:10.1186/s40249-016-0221-y)
Supplement: Additional file 1: — Multilingual abstracts in the five official working languages of the United Nations. (PDF 849 kb) [file 40249_2016_221_MOESM1_ESM.pdf]

Translation of the abstract into the five official working languages of the United Nations

العلاقة بين الملاريا وسوء التغذية لدى الأطفال دون الخامسة في منطقة شاشوغو، جنوبي إثيوبيا: دراسة حالة مقارنة

تيريف غون وفيساهيه لامانغو وإنديل إليسو وصاموئيل يوهانس وتاديل يوهانس

ملخص

نبذة: تمخضت الدراسات الأخيرة عن نتائج متضاربة بشأن علاقة الملاريا بزيادة خطر سوء التغذية أو نقصانه. ولهذا، فإن تقييم العلاقة بين هذين المرضين الوبيلين ضمن الفئات الأكثر ضعفًا، مثل الأطفال الذين تقل أعمارهم عن خمس سنوات (الأطفال دون الخامسة)، قد يؤدي إلى اكتشاف وسائل فعالة ومنخفضة التكلفة تمثل إضافة جديدة إلى الأساليب المطبقة حاليًا للوقاية من سوء التغذية في مناطق توطن الملاريا. ومن هذا المنطلق، أجريت هذه الدراسة من أجل تقييم العلاقة بين الملاريا وسوء التغذية فيما بين الأطفال دون الخامسة في منطقة موبوءة بدرجة عالية من انتقال عدوى الملاريا.

الأساليب: شملت الدراسة مقارنة بين أطفالٍ يعانون من سوء التغذية تتراوح أعمارهم بين 6 أشهر و59 شهرًا وأطفالٍ سليمي التغذية من نفس الفئة العمرية من حيث التعرض السابق للملاريا في منطقة شاشوغو، جنوبي إثيوبيا. وجرى استخدام استبيان موثق ومحدد البنية لجمع البيانات الاجتماعية والاقتصادية من منزل لآخر وأدوات أنثروبومترية لجمع البيانات السريرية. ومن ثم، جرى تحليل البيانات المجموعة عبر الإحصاء الوصفي والاستدلالي باستخدام برنامج EpiData (إبيداتا) لإدخال البيانات وبرنامج STATA (ستاتا) لتحليل البيانات.

النتائج: شارك 356 طفلًا دون الخامسة (89 يعانون من سوء التغذية و267 سليمي التغذية) في الدراسة. ووجِد أن التعرض السابق لعدوى المتصورات يعد مؤشرًا على وجود سوء تغذية لدى الأطفال دون الخامسة (*OR*=1.87, *P*=0.02 [*CI*=1.115–3.138]). كما وجِد أن الأطفال المنحدرين من أسر يقل دخلها الشهري عن 15 دولارًا أمريكيًا يكونون أكثر عرضة لسوء التغذية بواقع أربعة أضعاف ونصف (4.5) مقارنة بغيرهم من الأطفال (*OR*=0.422, *P*=0.001 [*CI*=0.181–0.978]).

الاستنتاج: أفادت هذه الدراسة بأن التعرض للمتصورة (جرثومة الملاريا) له تأثير كبير على الحالة التغذوية للأطفال، وأن العوامل الاجتماعية والديموغرافية، مثل دخل الأسرة، قد تلعب دورًا في تحديد إصابة الأطفال بسوء التغذية، بل يمكن أن تؤدي إلى زيادة الأمراض الناجمة عن سوء التغذية لدى الأطفال الذين يعيشون في مناطق توطن الملاريا. وتوصلت الدراسة إلى أنه يجب تعزيز التدخلات التي تستهدف مكافحة سوء التغذية باستراتيجيات للوقاية من الملاريا وخاصة في مناطق الانتقال السريع لهذا الوباء.

Translated from English version into Arabic by Ibrahim Abubakr, through

TRANSLATORS
WITHOUT BORDERS

埃塞俄比亚南部 Shashogo 区一项病例对照研究揭示的 5 岁以下儿童疟疾和营养不良的关系

Terefe Gone, FisehaLemango, Endale Eliso, Samuel Yohannes and TadeleYohannes

摘要

前言：近来的研究关于疟疾到底导致营养不良的风险增加还是减少出现了不一致的结果。因此，评估在脆弱人群（如 5 岁以下儿童）这两种严重疾病的相关性有助于在疟疾流行区预防营养不良方面发现新的、廉价且有效的措施。因此，开展本研究旨在评估某一疟疾高传播地区 5 岁以下儿童的疟疾和影响不良的相关性。
方法：本研究比较埃塞俄比亚南部 Shashogo 区的 6-59 个月大的营养不良儿童和正常儿童的既往疟疾患病情况。通过业已验证的结构性问卷收集收集家庭和社会经济数据，通过人体测量仪器测量临床数据。使用 EpiData 软件录入数据，使用 STATA 分析软件进行描述性分析和推断性分析。
结果：共有 356 名 5 岁以下儿童纳入研究，其中营养不良儿童 89 名，营养正常儿童 267 名。研究发现既往感染疟原虫是 5 岁以下儿童出现营养不良的预测指标（*P*=0.02 [*OR*=1.87, *CI*=1.115–3.138]）。来自月收入低于 15 美元家庭的儿童出现营养不良的几率比对照儿童高 4.5 倍（*P*=0.001 [*OR*=0.422, *CI*=0.181–0.978]）。
结论：本研究发现既往感染疟原虫对儿童营养状况有显著影响。此外，在疟疾流行区，社会人口因素如家庭收入也会影响儿童是否出现营养不良，并导致营养不良相关并发症的增加。因此，在高疟疾传播地区，需要通过疟疾防控措施来巩固营养不良防控措施的成果。

Translated from English version into Chinese by Men-Bao Qian, through

CHINESE CENTER FOR DISEASE CONTROL AND PREVENTION
NATIONAL INSTITUTE OF PARASITIC DISEASES

Le lien entre paludisme et malnutrition chez les enfants de moins de cinq ans, dans le district de Shashogo, en Éthiopie du Sud : une étude cas-témoin

Terefe Gone, FisehaLemango, Endale Eliso, Samuel Yohannes et TadeleYohannes

Résumé

Contexte : Des études récentes ont présenté des résultats contradictoires sur le lien entre le paludisme et une augmentation ou une diminution du risque de malnutrition. Par conséquent, l'étude de la relation entre ces deux maladies catastrophiques parmi les groupes les plus vulnérables, comme les enfants âgés de moins de cinq ans, pourrait conduire à la découverte de nouveaux outils peu coûteux et efficaces qui pourraient aider les méthodes actuelles de prévention de la malnutrition dans les zones impaludées. Cette étude a donc été menée afin d'évaluer la relation entre le paludisme et la malnutrition chez les enfants de moins de cinq ans, dans une zone qui connaît un degré élevé de transmission du paludisme.
Méthodes : L'étude consistait à comparer une exposition antérieure au paludisme d'enfants malnutris âgés de 6 à 59 mois et d'enfants bien nourris du même âge, dans le District de Shashogo, en Éthiopie du Sud. Un questionnaire structuré validé a permis de recueillir des données socioéconomiques en porte-à-porte et des instruments anthropométriques ont été utilisés pour les

données cliniques. Les données recueillies ont été analysées à l’aide de statistiques descriptives et inductives au moyen du logiciel de saisie EpiData et du logiciel d’analyse de données STATA.
Résultat : Un total de 356 enfants de moins de cinq ans (89 malnutris et 267 bien nourris) ont participé à l’étude. Une exposition antérieure à l’infection à *Plasmodium* s’est avérée être un facteur prédictif de la manifestation de la malnutrition chez les enfants de moins de cinq ans (P=0,02 [OU=1,87, CI=1,115–3,138]). Les enfants d’un ménage dont le revenu mensuel est inférieur à 15 USD étaient 4,5 fois plus susceptibles d’être malnutris par rapport aux autres enfants (P=0,001 [OR=0,422, CI=0,181–0978]).
Conclusion : Cette étude a révélé que l’exposition à *Plasmodium* a un impact significatif sur l’état nutritionnel des enfants. En outre, les facteurs sociodémographiques, tels que le revenu familial, pourraient jouer un rôle pour déterminer si les enfants sont malnutris ou pas et pourraient entraîner une morbidité accrue due à la malnutrition chez les enfants vivant dans des zones impaludées. Par conséquent, les interventions de contrôle de la malnutrition doivent être regroupées avec des stratégies de prévention du paludisme, en particulier dans les zones connaissant un degré élevé de transmission du paludisme.

Translated from English version into French by Patricia Sommer, through

TRANSLATORS
WITHOUT BORDERS

Связь между малярией и недоеданием среди детей, которым младше пяти лет в районе Shashogo Южной Эфиопии: одно случай-контрольное исследование.

Terefe Gone, FisehaLemango, Endale Eliso, Samuel Yohannes and TadeleYohannes

Реферат

Фон: Последние исследования показали такие противоречивые итоги как малярия связана с увеличивающим или снижающим риском недоедания. Таким образом, возможно найти экономические и эффективные меры для предотвращения недоедания в сфере эндемичных по малярии через оценивать связь между этими двумя катастрофическими заболеваниями среди наиболее уязвимых групп населения, например, среди детей, которым младше пяти лет. Следовательно, данное исследование напраленно на оценки связи между малярией и недоеданием среди детей, которым младше пяти лет в одном районе с высокой степеней передачи малярии.
Методы: Данное исследование включает в себя сравнение по недоеданию среди детей, которым 6-59 месяцем и питаемых детей в районе Shashogo Южной Эфиопии, чтобы выяснить последние подверженности малярии. С помощью проверенных структурированных анкетов собирать социально-экономические данные по домам и антропометрические приборы для клинических данных. Собиранные данные были анализированны записью программного обеспечения EpiData и программным обеспечением для анализа данных для описательных и выведенных статистик.

Результатаы: Всего 356 детей, которым младше пяти лет приняли участие в этом исследовании. Предыдущее подвержение к Плазмодии инфекции было найдено как предиктор для проявления недоедания среди детей, которым младше пяти лет (*P*=0.02 [*OR*=1.87, *CI*=1.115–3.138]). Дети в семье, у которой домохозяйства с ежемесячным доходом менее $ 15 получают более чем в 4.5 раза шанса страдать недоеданией по сравнению с другими детьми (*P*=0.001 [*OR*=0.422, *CI*=0.181–0.978]).
Заключение: Это исследование показало, что воздействие Плазмодия оказывает существенное влияние на статус питания детей. Кроме того, социально-демографические факторы, как доходы семьи играет важную роль в определении того, является ли ребенок недоедает или не может привести к повышению заболеваемости вследствие недоедания у детей, проживающих в эндемичных по малярии районах. Так, недоедание меры контроля должны быть объединены с помощью стратегий профилактики малярии, особенно в районах с высоким уровнем передачи малярии.

Translated from English version into Russian by Hao-Qi Zhang, through

La conexión entre la malaria y la desnutrición entre niños menores de cinco años en el Distrito Shashogo del sur de Etiopía: un estudio de control de caso

Terefe Gone, Fiseha Lemango, Endale Eliso, Samuel Yohannes y TadeleYohannes

Resumen
Antecedentes: Estudios recientes han arrojado resultados contradictorios respecto a la conexión entre la malaria y un riesgo mayor o menor de desnutrición. Por lo tanto, evaluar la relación entre estas dos enfermedades desastrosas en los grupos más vulnerables, como el de los niños menores de cinco años de edad, puede conducir al descubrimiento de nuevos apoyos efectivos y de bajo costo para los métodos actuales de prevención de desnutrición en zonas de malaria endémica. Por lo mismo, este estudio fue realizado para evaluar la relación entre la malaria y la desnutrición entre niños menores de cinco años en una zona con un alto grado de transmisión de malaria.
Métodos: El estudio implicó la comparación de niños desnutridos de entre 6 y 59 meses de edad y niños debidamente alimentados de la misma edad según su previa exposición a la malaria en el Distrito Shashogo, en el sur de Etiopía. Se utilizó un cuestionario estructurado validado para recolectar información socioeconómica de casa en casa e instrumentos antropométricos para datos clínicos. La información recaudada se analizó utilizando estadísticas descriptivas e inferenciales mediante el software de introducción de datos EpiData y el software STATA de análisis de datos.
Resultados: Participaron en el estudio un total de 356 niños menores de cinco años (89 desnutridos y 267 bien alimentados). Se descubrió que la exposición previa a la infección de *Plasmodium* era un indicador de la manifestación de desnutrición en niños menores de cinco años (*P*=0.02 [*RM*=1.87, *IC*=1.115–3.138]). Los niños de hogares con un ingreso mensual menor a los 15 dólares (USD)

tenían un 4.5 más de probabilidades de desnutrición en comparación con los otros niños (*P*=0.001 [*RM*=0.422, *IC*=0.181–0.978]).
Conclusión: Este estudio estableció que la exposición a la infección de *Plasmodium* tiene un impacto significativo sobre el estado nutricional de los niños. Además, factores socio-demográficos, como el ingreso familiar, pueden contribuir a determinar si los niños están o no desnutridos, y pueden conducir a una mayor morbilidad debido a la desnutrición en niños que viven en zonas donde la malaria es endémica. Por lo tanto, deben consolidarse intervenciones de control de la desnutrición con estrategias de prevención de la malaria, particularmente en zonas con una alta transmisión de la misma.

Translated from English version into Spanish by Adriana Díaz Enciso, through

TRANSLATORS
WITHOUT BORDERS
